# 

**Published:** 1847-01

**Authors:** 


### B[illegible]ISH AND FOREIGN

### MEDICAL REVIEW

OR

### QUARTERLY JOURNAL

OF

### PRACTICAL MEDICINE AND SURGERY

---

EDITED BY

JOHN FORBES M.D. F.R.S. F.G.S.

---

S.G.O.L. INDEXED

VOL. XXIII.

JANUARY—APRIL 1847

Surgeon Genl's Office LIBRARY. 34546 Washington D.C.

LONDON

JOHN CHURCHILL PRINCES STREET SOHO

MDCCCXLVII.

W
1
BR 34

C AND J. ADLARD, PRINTERS, BARTHOLOMEW CLOSE.

### THE BRITISH AND FOREIGN MEDICAL REVIEW.

**No. XLV.—January, 1847.**

### Part First.—Analytical and Critical Reviews.

PAGE

1. Gross, Gely, and Dieffenbach on Wounds of the Intestines, &c. . 1

2. Professor Lanza's Positive Nosology and Positive Medicine . . 37

3. Dr. Gross's Elements of Pathological Anatomy . . . 63

4. French Military Memoirs in Medicine, Surgery, and Pharmacy . . 73

5. Renouard, Emiliani, Leupoldt on Medical History.—Medicine: its Past, Present, and Future . . . . . . . . 85

6. Dr. Geddes's Clinical Illustrations of the Diseases of India . . 106

7. Dr. Wright on Saliva . . . . . . . 129

8. M. Burggraeve on Histology applied to Physiology and Pathology . 136

9. Dr. Parkes on the Dysentery and Hepatitis of India . . . 146

10. Malgaigne's Operative Surgery, by Brittan . . . . 155

11. Boussingault, Liebig, Thomson on the Food of Animals . . 157

12. Mr. Marshall on Military Punishments . . . . 163

13. Dr. Latham's Lectures on Diseases of the Heart . . . 169

14. Dr. Marshall Hall's Observations in Medicine . . . 185

15. Dr. Moreau's Psychological Studies on Hachisch and on Mental Derangement 217

### Part Second.—Bibliographical Notices.

1. Dr. Underwood's Treatise on the Diseases of Children, by Dr. Davies . 237

2. Mr. Griffiths's Chemistry of the Four Seasons . . . 238

3. Dr. Ashwell on the Diseases of Women . . . . 239

4. Dr. Guy on the Health of Towns . . . . . 240

5. Dr. M'Connell Reed on Fever . . . . . 241

6. Sir B. Brodie, Dr. Guy, Mr. Paget on Medical Education . . 242

7. Dr. Royle's Manual of Materia Medica . . . . 244

8. Dr. Carpenter on Human Physiology . . . . 245

9. Mr. Wharton Jones on Ophthalmic Medicine and Surgery . . 246

10. Mr. Grove's Lectures on Physical Forces . . . . 247

11. Dr. Golding Bird on Urinary Deposits . . . . 248

12. Descriptive Catalogue of the Anatomical Museum of St. Bartholomew's Hospital 249

13. Mr. Paget's Records of Harvey . . . . . ib.

14. Mr. Ellis's Lectures on Clinical Surgery . . . . 250

15. Dr. Day's Vogel's Anatomy . . . . . ib.

16. Works published by the Sydenham Society: Dr. Swaine's Hasse's Anatomical Description of the Diseases of the Organs of Respiration; Dr. Adams's Paulus Ægineta; Mr. Gulliver's Works of Hewson . . . . 251

17. Mr. E. Wilson on the Skin . . . . . ib.

18. Dr. Symonds on Knowledge . . . . . 252

19. Mr. Hassall's Microscopic Anatomy . . . . ib.

20. M. Littré's Hippocrates . . . . . 253

21. Dr. Combe on National Education . . . . 254

22. Dr. Griffith's Practical Manual . . . . . ib.

23. Mr. Birkett's Von Behr's Handbook of Human Anatomy . . 255

24. Dr. Walker on Fractures . . . . . . 256

### Part Third.—Natural History and Treatment of Diseases.

1. Dr. Combe on the Observation of Nature in the Study and Treatment of Diseases 257
2. Illustrations of the Vis Medicatrix . . . . . 265
3. Mr. Stallard on the Treatment of Fever by Cold Water . . 269

### Part Fourth.—Original Reports and Memoirs.

Dr. West's Report on the Progress of Practical Medicine in the Departments of Midwifery and the Diseases of Women and Children, in the Years 1845-6 . 277

On a New Means of rendering Surgical Operations painless . . . 309

---

**No. XLVI WILL BE PUBLISHED ON THE 1st OF APRIL, 1847.**

---

Communications, Journals, *and* Books for Review, *to be forwarded (Carriage paid) to the care of* Mr. John Churchill, Princes Street, Soho; *or direct to the* Editor, 12, Old Burlington Street, London.

